# Supplementary material for: Histological hallmarks and role of Slug/PIP axis in pulmonary hypertension secondary to pulmonary fibrosis
Source: EMBO Mol Med. 2019 Aug 29;11(9):e10061. doi: 10.15252/emmm.201810061 (PMC6728601; doi:10.15252/emmm.201810061)
Supplement: Supplementary file 1 — Appendix [file EMMM-11-e10061-s001.pdf]

Appendix

Table of content:

Appendix Table S1: Patients characteristics

Appendix Table S2: Expression of Slug transcriptional targets genes

Appendix Table S3: Slug transcriptional targets annotated for proliferation

Appendix Table S4: Slug transcriptional targets annotated for extracellular matrix

Appendix Table S5: Statistics

Appendix Table S1

	Diagnostic	Age	Sex	mPAP (mmHg)	CO (L.min^{-1})	PVR (WU)	Medication	6MWD (m)	FVC (L)	FVC (% predicted)	FEV1(L)	FEV1 (%predicted)	FEV1/FVC	DLCO (mL/min/mmHg)	DLCO (% predicted)	Oxygen saturation	Oxygen use (L)
**Ctrl**	Pneumothorax	20	M	n/a	n/a	n/a	n/a	n/a	n/a	n/a	n/a	n/a	n/a	n/a	n/a	n/a	n/a
	Pneumothorax	21	M	n/a	n/a	n/a	n/a	n/a	n/a	n/a	n/a	n/a	n/a	n/a	n/a	n/a	n/a
	Pneumothorax	34	M	n/a	n/a	n/a	n/a	n/a	n/a	n/a	n/a	n/a	n/a	n/a	n/a	n/a	n/a
	Healthy lung	73	M	n/a	n/a	n/a	n/a	n/a	n/a	n/a	n/a	n/a	n/a	n/a	n/a	n/a	n/a
**PAH**	iPAH	48	F	65	5.7	n/a	Trep.<br>Maci.<br>Amb.<br>Tada.	390	n/a	n/a	n/a	n/a	n/a	n/a	n/a	n/a	n/a
	iPAH	24	F	65	4.44	n/a	Trep.<br>Amb.<br>Tada.	n/a	n/a	n/a	n/a	n/a	n/a	n/a	n/a	n/a	n/a
	iPAH	49	F	65	n/a	n/a	n/a	n/a	n/a	n/a	n/a	n/a	n/a	n/a	n/a	n/a	n/a
	iPAH	55	F	55	8.97	4.8	Epop.<br>Tada.	475	n/a	n/a	n/a	n/a	n/a	n/a	n/a	n/a	n/a
	iPAH	42	F	78	4.2	15.9	Epop.<br>Maci.<br>Tada	96	n/a	n/a	n/a	n/a	n/a	n/a	n/a	n/a	n/a
	iPAH	34	F	56	2.87	n/a	Trep.<br>Maci.<br>Tada	210	n/a	n/a	n/a	n/a	n/a	n/a	n/a	n/a	n/a
	iPAH	50	F	59	6.65	6.61	Trep.<br>Maci.<br>Tada	90	n/a	n/a	n/a	n/a	n/a	n/a	n/a	n/a	n/a
**PF**	iPF	68	M	23	4.3	1.9	-	111	1.57	38.5	1.41	44.9	89	n/a	29	94	15
	UIP	51	M	23	8.3	n/a	-	210	1.87	36.9	1.62	41.2	87	7.4	22.1	99	6
	iPF	64	M	24	6.1	2.3	-	120	3.68	70	3.22	90	88	6	20	94	2
	iPF	64	M	20	6.2	1.61	-	249	3.09	58	2.48	62.1	80	9.05	26.8	96	3
	NSIP	42	F	24	5.28	3.03	-		2.52	71	2.26	77	90	5.3	17	100	3
	UIP	62	M	20	4.4	1.77	-	430	n/a	n/a	n/a	n/a	n/a	n/a	n/a	97	6
	CHP	42	F	20	5.67	3.18	-		0.86	21.2	0.79	24	91	4.3	17	96	6
	iPF	72	M	24	5.8	3	-	240	1.87	46	1.56	53	84	n/a	n/a	91	4

	iPF	72	M	22	5.4	n/a	Pirf.	120	3.85	88	2.94	91	76	9.86	33.6	92	0
	iPF	64	M	16	5.17	n/a	-	208	2.01	42	1.67	46	83	9.05	32	94	6
	iPF	66	F	13	2.6	n/a	-	124	0.93	37	0.66	34	71	3.6	27	100	4
	UIP	54	F	23	4	3.5	-	156	2.01	42	1.67	46	83	2.64	11.9	100	4
	UIP	58	F	10	4.05	n/a	-	n/a	0.88	31	0.74	33	84	7.66	39	99	2
	UIP	68	M	23	5.76	2.94	Vals.	465	2.57	54	2.15	61	84	5.36	17.4	93	2
**PF-PH**	UIP/PH	73	M	27	n/a	2.6	-	475	2.17	55.2	1.62	56.3	74	9.04	33.4	100	3
	iPF/PH	66	M	27	5.47	3.1	Pirf.	258	4.25	93.1	2.85	83.7	67	12.1	42	96	3
	iPF/PH	71	M	26	5.36	n/a	-	327	2.47	56.4	2.24	69.5	90	10.1	40	97	3
	iPF/PH	65	F	32	4.95	3.6	Losa.	128	1.64	53	1.54	65	94	5.6	25	95	5
	iPF/PH	68	F	26	4.7	n/a	-	60	0.95	34	0.87	41	91	n/a	n/a	93	6
	iPF/PH	66	F	36	3.6	5.7	Sild.	209	1.17	42	1.02	48	87	5.54	26.6	100	6
	iPF/PH	65	M	26	4.1	4	-	24	2.16	53	1.95	69	90	6.16	22	97	8
	iPF/PH	64	F	26	3.5	n/a	-	n/a	0.66	26.6	0.62	32.8	94	n/a	n/a	99	6
	UIP/PH	65	F	30	4	5.5	-	240	1.53	49	1.37	57	89	4.14	18.2	100	4
	UIP/PH	64	F	44	4.2	7.6	Trep. Tada.	195	1.43	52	1.16	55	81	3.38	16.5	99	6
	CHP/PH	34	M	37	7.1	n/a	-	n/a	1.01	18.2	0.76	16.6	75	8.25	22	95	2
	UIP/PH	66	M	47	3.83	6.54	-	n/a	1.42	33	1.23	37	86	2.7	n/a	96	6
	iPF/PH	61	M	32	4.6	n/a	Pirf. Sild.	137	2.37	63	2.24	76	94	8.5	33	99	8
	UIP/NSIP/PH	57	M	35	5.6	5.36	Tada.	90	2.98	70.5	2.59	78	87	4.1	13.4	93	3

Appendix table S1: Patients Characteristics. Abbreviations: iPAH: idiopathic pulmonary arterial hypertension; iPF: idiopathic pulmonary fibrosis; UIP: usual interstitial pneumonia; PH: pulmonary hypertension; CHP: chronic hypersensitivity pneumonitis; NSIP: nonspecific interstitial pneumonia. Trep. Treprostinil; Maci. Macitentan; Amb. Ambrisentan; Tada. Tadalafil; Epop. Epoprostenol; Sild. Sildenafil; Pirf. Pirfenidone; Vals. Valsartan; Losa. Losartan

Appendix Table S2 Expression of Slug transcriptional target genes

Entrez gene	Symbol	Fold Change	Adjusted p-value
199920	C1ORF168	1.71	1.6E-02
3866	KRT15	1.69	4.4E-02
4585	MUC4	1.67	4.2E-02
5304	PIP	1.63	3.9E-02
72	ACTG2	1.57	1.1E-02
158798	AKAP14	1.57	1.7E-02
1999	ELF3	1.53	4.9E-03
284434	NWD1	1.49	2.6E-02
90827	ZNF479	1.44	1.17E-08
338322	NLRP10	1.43	5.85E-11
384	ARG2	1.42	1.1E-03
2302	FOXJ1	1.42	7.2E-03
9289	ADGRG1	1.39	2.5E-03
51364	ZMYND10	1.38	1.7E-02
3872	KRT17	1.35	4.1E-02
2524	FUT2	1.33	4.0E-02
388611	CD164L2	1.32	1.65E-05
9013	TAF1C	1.31	2E-10
5578	PRKCA	1.29	8.13E-07
5498	PPOX	1.29	3.7E-03
9536	PTGES	1.28	3.E-047
60673	ATG101	1.28	8.0E-04
414332	LCN10	1.27	8.18E-06
91860	CALML4	1.27	1.1E-03
375307	CATIP	1.27	1.89E-05
254050	LRRC43	1.27	3.8E-02
225689	MAPK15	1.26	3.5E-03
4142	MAS1	1.26	1.48E-05
3485	IGFBP2	1.25	1.1E-02
122769	LRR1	1.25	3.35E-05
9892	SNAP91	1.25	1.87E-06

1081	CGA	1.24	1.28E-05
3782	KCNN3	1.24	2.4E-03
1791	DNTT	1.24	8.19E-06
3753	KCNE1	1.24	1.3E-05
6625	SNRNP70	1.23	3.89E-05
126328	NDUFA11	1.23	5.66E-05
2571	GAD1	1.23	5.56E-04
7712	ZNF157	1.22	4.4E-04
55971	BAIAP2L1	1.22	3.3E-02
10391	CORO2B	1.21	4.9E-03
9369	NRXN3	1.21	4.6E-02
9123	SLC16A3	1.21	6.6E-03
8100	IFT88	1.21	4.4E-02
51764	GNG13	1.21	5.32E-06
80781	COL18A1	1.21	2.17E-04
8938	BAIAP3	1.21	2.2E-02
4588	MUC6	1.21	5.4E-03
51265	CDKL3	1.21	6.87E-05
7355	SLC35A2	1.21	2.75E-05
283927	NUDT7	1.21	2.4E-02
4357	MPST	1.20	5.45E-05
221002	RASGEF1A	1.20	9.23E-08
2779	GNAT1	1.20	8.37E-06
27324	TOX3	1.20	4.0E-02
7441	VPREB1	1.20	2.7E-04
81857	MED25	1.20	6.87E-05
29948	OSGIN1	1.20	9.33E-05

Appendix Table S3 Slug transcriptional targets annotated for proliferation

Entrez gene	Gene Symbol	Fold Change	Adjust p-value
4585	MUC4	1.66	4.2E-03
5304	PIP	1.62	3.9E-02
3872	KRT17	1.35	4.1E-02
5498	PPOX	1.295	3.7E-03
4142	MAS1	1.26	1.48E-05
3485	IGFBP2	1.25	1.1E-02
2779	GNAT1	1.20	8.37E-06
3868	KRT16	1.18	5.8E-04
8425	LTBP4	1.17	4.7E-02
8764	TNFRSF14	1.16	1.0E-02
6725	SRMS	1.13	4.5E-03
995	CDC25C	1.12	4.1E-02
2068	ERCC2	1.10	4.3E-02
5333	PLCD1	1.10	1.6E-02
11334	TUSC2	1.09	3.6E-02
283870	BRICD5	1.09	4.6E-03

Appendix Table S4 Slug transcriptional targets annotated for extracellular matrix

Entrez gene	Gene Symbol	Fold Change	Adjusted p-value
4585	MUC4	1.66	4.2E-02
5304	PIP	1.62	3.9E-02
72	ACTG2	1.56	1.1E-02
5498	PPOX	1.29	3.7E-03
414332	LCN10	1.27	8.2E-06
225689	MAPK15	1.26	3.5E-04
4142	MAS1	1.26	1.5E-05
3485	IGFBP2	1.25	1.1E-02
1081	CGA	1.24	1.2E-05
80781	COL18A1	1.21	2.1E-04
4588	MUC6	1.21	5.4E-03
7441	VPREB1	1.20	2.7E-04
79258	MMEL1	1.17	2.4E-04
5020	OXT	1.17	1.9E-03
1297	COL9A1	1.17	2.6E-04
8425	LTBP4	1.17	4.7E-02
144453	BEST3	1.17	1.9E-11
5657	PRTN3	1.15	2.6E-03
2316	FLNA	1.14	1.8E-02
5364	PLXNB1	1.14	3.6E-02
386653	IL31	1.14	5.4E-03
79174	CRELD2	1.14	1.6E-02
27098	CLUL1	1.14	2.3E-03
1463	NCAN	1.14	5.5E-03
4838	NODAL	1.13	0.014
81	ACTN4	1.12	0.006
64806	IL25	1.12	0.005
653808	ZG16	1.11	0.009
3562	IL3	1.10	0.020
23436	CELA3B	1.10	0.033
1775	DNASE1L2	1.10	0.027
283870	BRICD5	1.09	0.004

3565	IL4	1.08	0.040
2323	FLT3LG	1.07	0.030
84639	IL1F10	0.91	0.043
80740	LY6G6C	0.89	0.018
124912	SPACA3	0.87	0.007
89790	SIGLEC10	0.81	0.001

Appendix Table S5: Statistics

Figure	Statistical Test	Comparison	p-value
Fig. 1A	ANOVA (One-way)	Ctrl vs. PF	<0.0001
		Ctrl vs. PAH	>0.9999
		Ctrl vs. PFPH	<0.0001
		PF vs. PAH	<0.0001
		PF vs. PFPH	>0.9999
		PAH vs. PFPH	<0.0001
Fig 1B	T-test (Unpaired; two-tail)	PF vs. PFPH	0.842
Fig 1C	T-test (Unpaired; two-tail)	PF vs. PFPH	0.8251
Fig 1D	T-test (Unpaired; two-tail)	PF vs. PFPH	0.4316
Fig 1E	ANOVA (One-way)	Ctrl vs. PF	<0.0001
		Ctrl vs. PAH	<0.0001
		Ctrl vs. PFPH	<0.0001
		PF vs. PAH	0.0001
		PF vs. PFPH	<0.0001
		PAH vs. PFPH	0.889
Fig 1F	ANOVA (two-way)	Non fibrosis	0.0006
		Mild fibrosis	0.0003
		Severe Fibrosis	<0.0001
Fig 1G	ANOVA (One-way)	PF vs PF-PH non fibrosis	<0.0001
		PF vs PF-PH fibrosis	<0.0001
	ANOVA (two-way)	PF fibro vs non-fibro	0.0028
		PF-PH fibro vs. non-fibro	0.0005
Fig 2A	T-test (Unpaired; two-tail)	PF vs. PFPH (EC)	0.0122
		PF vs. PFPH (SMC)	0.0012
Fig 2B	T-test (Unpaired; two-tail)	PF vs. PFPH	0.0184
Fig 2C	T-test (Unpaired; two-tail)	PF vs. PFPH	0.0276

Fig 2D	T-test (Unpaired; two-tail)	PF vs. PFPH (EpC)	0.4673
		PF vs. PFPH (Fib)	0.9124
		PF vs. PFPH (Macro Φ)	0.01
		PF vs. PFPH (EC)	0.7689
		PF vs. PFPH (SMC)	#N/A
Fig 2E	ANOVA (one-way)	PF vs. PF-PH (non fibro)	0.0225
		PF vs. PF-PH (Fibro)	0.0287
	ANOVA (two-way)	PF	0.0363
		PF-PH	0.0019
Fig 3D	T-test (Unpaired; two-tail)	PF vs. PFPH	0.0035
Fig 3E	T-test (Unpaired; two-tail)	PF vs. PFPH	0.0125
Fig 3F	T-test (Unpaired; two-tail)	PF vs. PFPH	0.0104
Fig 3G	ANOVA (one-way)	Ctrl vs 12.5	0.689
		Ctrl vs 25	0.2456
Fig 3H	ANOVA (one-way)	Ctrl vs 12.5	0.05
		Ctrl vs 25	0.04
Fig 3I	ANOVA (one-way)	Ctrl vs 12.5	0.0214
		Ctrl vs 25	0.0027
Fig 3J	ANOVA (one-way)	Ctrl vs 12.5	0.0097
		Ctrl vs 25	0.0005
Fig 4A	T-test (Unpaired; two-tail)	Ctrl vs. Bleo 2weeks	<0.0001
Fig 4B	T-test (Unpaired; two-tail)	Ctrl vs. Bleo 2weeks	<0.0001
Fig 4C	T-test (Unpaired; two-tail)	Ctrl vs. Bleo 2weeks	<0.0001
Fig 4D	T-test (Unpaired; two-tail)	Ctrl vs. Bleo 2weeks	<0.0001
Fig 4F	ANOVA (One-way)	CTRL vs. Bleo	0.9715
		CTRL vs. MCT	0.013
		CTRL vs. Bleo-MCT	<0.0001
		Bleo vs. MCT	0.0267
		Bleo vs. Bleo-MCT	<0.0001
		MCT vs. Bleo-MCT	0.0054
Fig 4G	ANOVA (One-way)	CTRL vs. Bleo	0.2128
		CTRL vs. MCT	0.0097
		CTRL vs. Bleo-MCT	0.0005
		Bleo vs. MCT	0.4677
		Bleo vs. Bleo-MCT	0.0457

		MCT vs. Bleo-MCT	0.6417
Fig 4H	ANOVA (One-way)	CTRL vs. Bleo	<0.0001
		CTRL vs. MCT	0.3889
		CTRL vs. Bleo-MCT	<0.0001
		Bleo vs. MCT	<0.0001
		Bleo vs. Bleo-MCT	0.8189
		MCT vs. Bleo-MCT	<0.0001
Fig 4I	ANOVA (One-way)	CTRL vs. Bleo	0.0025
		CTRL vs. MCT	0.0106
		CTRL vs. Bleo-MCT	0.0219
		Bleo vs. MCT	0.933
		Bleo vs. Bleo-MCT	0.801
		MCT vs. Bleo-MCT	0.989
Fig 4J	ANOVA (One-way)	CTRL vs. Bleo	0.0066
		CTRL vs. MCT	0.8347
		CTRL vs. Bleo-MCT	0.0011
		Bleo vs. MCT	0.0452
		Bleo vs. Bleo-MCT	0.8903
		MCT vs. Bleo-MCT	0.0089
Fig 4K	ANOVA (One-way)	CTRL vs. Bleo	0.0049
		CTRL vs. MCT	0.9976
		CTRL vs. Bleo-MCT	0.0309
		Bleo vs. MCT	0.0078
		Bleo vs. Bleo-MCT	0.8608
		MCT vs. Bleo-MCT	0.0466
Fig 4L	ANOVA (One-way)	CTRL vs. Bleo	0.8909
		CTRL vs. MCT	<0.0001
		CTRL vs. Bleo-MCT	<0.0001
		Bleo vs. MCT	0.0001
		Bleo vs. Bleo-MCT	<0.0001
		MCT vs. Bleo-MCT	0.9892
Fig 4M	ANOVA (One-way)	Bleo vs. Bleo-MCT (non fibro)	<0.0001
		Bleo vs. Bleo-MCT (fibro)	<0.0001
	ANOVA (two-way)	Bleo	0.0464
		Bleo-MCT	0.2278

Fig 5A	T-test (Unpaired; two-tail)	Bleo vs. Bleo-MCT (EC)	0.0707
		Bleo vs. Bleo-MCT (SMC)	0.0081
Fig 5B	ANOVA (One-way)	CTRL vs. Bleo	0.6646
		CTRL vs. MCT	0.6206
		CTRL vs. Bleo-MCT	0.2295
		Bleo vs. MCT	0.9998
		Bleo vs. Bleo-MCT	0.0176
		MCT vs. Bleo-MCT	0.0148
Fig 5C	ANOVA (One-way)	CTRL vs. Bleo	0.9973
		CTRL vs. MCT	0.9999
		CTRL vs. Bleo-MCT	0.0095
		Bleo vs. MCT	0.9931
		Bleo vs. Bleo-MCT	0.006
		MCT vs. Bleo-MCT	0.0113
Fig 5D	T-test (Unpaired; two-tail)	Bleo vs. Bleo-MCT (EpC)	0.7466
		Bleo vs. Bleo-MCT (Fib)	0.8805
		Bleo vs. Bleo-MCT (Macro Φ)	0.0266
		Bleo vs. Bleo-MCT (EC)	#N/A
		Bleo vs. Bleo-MCT (SMC)	#N/A
Fig 5E	T-test (Unpaired; two-tail)	Bleo vs. Bleo-MCT	0.6278
Fig 5F	ANOVA (One-way)	Bleo vs. Bleo-MCT (non fibro)	0.05
		Bleo vs. Bleo-MCT (fibro)	0.0326
	ANOVA (two-way)	Bleo	0.0498
		Bleo-MCT	0.0375
Fig 6A	T-test (Unpaired; two-tail)	Bleo vs. Bleo-MCT	0.031
Fig 6B	T-test (Unpaired; two-tail)	Bleo vs. Bleo-MCT	0.0016
Fig 6C	T-test (Unpaired; two-tail)	Bleo vs. Bleo-MCT	0.0388
Fig 7B	T-test (Unpaired; two-tail)	Si-Scrm vs Si-Slug	0.0007
Fig 7C	T-test (Unpaired; two-tail)	Si-Scrm vs Si-Slug	0.0204
Fig 7D	T-test (Unpaired; two-tail)	Si-Scrm vs Si-Slug	0.0024
Fig 7E	T-test (Unpaired; two-tail)	Si-Scrm vs Si-Slug	0.0024
Fig 7F	T-test (Unpaired; two-tail)	Si-Scrm vs Si-Slug	0.7385
Fig 7G	T-test (Unpaired; two-tail)	Si-Scrm vs Si-Slug	0.4271
Fig 7H	T-test (Unpaired; two-tail)	Si-Scrm vs Si-Slug	0.8538
Fig 7I	T-test (Unpaired; two-tail)	Si-Scrm vs Si-Slug	0.1764

Fig 7J	T-test (Unpaired; two-tail)	Si-Scrm vs Si-Slug	0.0041
Fig 7K	ANOVA (One-way)	Si-Scrm vs Si-Slug (non fibro)	0.003
		Si-Scrm vs Si-Slug (fibro)	0.0059
	ANOVA (two-way)	Si-Scrm	0.0318
		Si-Slug	0.0122
Fig 7L	T-test (Unpaired; two-tail)	Si-Scrm vs Si-Slug (SMC)	<0.0001
		Si-Scrm vs Si-Slug (EC)	0.0009
Fig 8A	T-test (Unpaired; two-tail)	Si-Scrm vs Si-Slug	<0.0001
Fig 8B	T-test (Unpaired; two-tail)	Si-Scrm vs Si-Slug	0.5324
Fig 8C	ANOVA (One-way)	Si-Scrm vs Si-Slug (non fibro)	0.0035
		Si-Scrm vs Si-Slug (fibro)	0.005
	ANOVA (two-way)	Si-Scrm	0.0162
		Si-Slug	0.026
Fig 8D	T-test (Unpaired; two-tail)	Si-Scrm vs Si-Slug	0.0028
Fig 8E	T-test (Unpaired; two-tail)	Si-Scrm vs Si-Slug	0.0003
Fig 8F	T-test (Unpaired; two-tail)	Si-Scrm vs Si-Slug	0.0024
